# Do Endangered Glacial Relicts Have a Chance for Effective Conservation in the Age of Global Warming? A Case Study: *Salix lapponum* in Eastern Poland

**DOI:** 10.3390/biology14010019

**Published:** 2024-12-28

**Authors:** Michał Arciszewski, Magdalena Pogorzelec, Marzena Parzymies, Urszula Bronowicka-Mielniczuk, Tomasz Mieczan

**Affiliations:** 1 Department of Hydrobiology and Protection of Ecosystems, University of Life Sciences in Lublin, Dobrzańskiego 37, 20-262 Lublin, Poland; michal.arciszewski@up.lublin.pl (M.A.); magdalena.pogorzelec@up.lublin.pl (M.P.); tomasz.mieczan@up.lublin.pl (T.M.); 2 Institute of Horticultural Production, University of Life Sciences in Lublin, Głęboka 28, 20-612 Lublin, Poland; 3 Department of Applied Mathematics and Computer Science, University of Life Sciences in Lublin, Głęboka 28, 20-612 Lublin, Poland; urszula.bronowicka@up.lublin.pl

**Keywords:** climate change, downy willow, oxidative stress, temperature stress, translocation

## Abstract

The abiotic stresses to which plants are exposed, especially in times of climate change, can result in the disruption of natural plant physiological processes. Sudden atmospheric phenomena may increase the risk of failure in protecting rare and extinction-threatened plant species by translocation. This study aimed to determine the effect of extreme ambient temperatures on the condition and physiological response of *Salix lapponum* plantlets used for their reintroduction into the natural habitat. *Salix lapponum* plants obtained by micropropagation methods at different stages of growth under laboratory conditions were subjected to a biological experiment. Plants were exposed for 12 h to temperature extremes (0 °C and 30 °C), after which the values of selected markers of the biochemical response were determined, such as photosynthetic pigments and anthocyanin content, guaiacol peroxidase and catalase activity, the presence of ROS and the RWC value. The study showed that plants at early growth stages were sensitive to low-temperature stress. In contrast, older ones showed a stronger response to high temperature, marked by an increased anthocyanin content and guaiacol peroxidase activity. It was also found that a short exposure to temperature extremes did not change the photosynthetic pigment content or catalase activity. The results of the study may be an important indication for the optimization of plant acclimatization methods in the process of their active protection by species translocation.

## Simple Summary

One of the challenges currently facing the conservation of endangered plant species is adapting the methods to the changing climate and increasing global warming. This problem is primarily linked to insufficient knowledge of whether and how quickly individuals of these species can adapt to changes in their natural habitats. Thus, it is difficult to predict whether their reintroduction into a natural environment will be successful. To come a little closer to answering this question, experiments were carried out on rare *Salix lapponum* plants grown in the growing room to determine how the plants respond to the stress of short-term exposure to extreme temperatures in the environment. It was found that the youngest plants were the most sensitive to low temperatures, while older ones reacted strongly to heat stress. This was indicated by an increased anthocyanin content in plant tissues and guaiacol peroxidase enzyme activity, which proved to be the most informative markers among the several used ones. The knowledge of the response of *Salix lapponum* to heat stress will help optimize plant acclimatization methods and perhaps contribute to the effective conservation of this valuable species of wetland flora in eastern Europe.

## 1. Introduction

Sudden atmospheric events, which are a direct consequence of global warming, pose an increasing threat to both humans and natural ecosystems. Extreme weather phenomena are predicted to become more frequent and intense [1,2]. In addition, periods of drought and heat waves repeated at short intervals will exacerbate the economic crisis and threaten the natural ecosystems [3]. These phenomena may not only lead to habitat degradation but also directly and indirectly contribute to species extinction, especially for those species with poor adaptive capacity [4,5].

As sedentary organisms, plants are directly exposed to changes in the environmental conditions and have to adapt to stressful stimuli quickly. Temperature, drought, salinity, or intense lighting affect plant growth and development. When any of these factors exceed the optimum tolerance value for a given species, plants activate a series of complex defense responses that include changes in gene expression and morphogenesis processes [6,7]. Mild stress, although it may slow plant growth, contributes to the activation of adaptive mechanisms. Intense and sudden stress, however, often leads to severe damage, even death [8].

Environmental stress induces the formation of reactive oxygen species (ROS) in plants, such as singlet oxygen (^1^O_2_), superoxide anion (O_2_−), and the most common hydrogen peroxide (H_2_O_2_). ROS disrupt plant homeostasis, can damage practically all cellular macromolecules, including DNA, and inhibit de novo protein synthesis [6]. Stress induced by both low and high temperatures also leads to multistage damage to the photosynthetic apparatus, particularly photosystem II (PS II), which results in reduced photosynthetic capacity, inhibited growth, and reduced productivity [9,10]. Enzymes such as superoxide dismutase (SOD), catalase (CAT), guaiacol peroxidase (GPOX), or ascorbate peroxidase (APX) play a key role in protecting plants from the effects of heat stress. They are involved in neutralizing ROS, reducing cell damage, and supporting plant survival [11].

There are lot of data on plant responses to stress factors, such as temperature changes, water availability, or pollution, but most of them, however, focus on cultivated or commercial plants [8,12]. In contrast, few data have been published on this topic for wildlife species, which represent ecological rather than economic value and are important for the biodiversity and the stability of natural ecosystems.

Active conservation of plant species threatened with extinction using translocation methods encounters many challenges in the conditions of current climate change. Many authors discuss the factors that may have a significant impact on the effects of reintroduction or population strengthening (knowledge of the biology and ecology of the species, selection of appropriate starting plant material, selection of appropriate habitat, etc.) and describe how to measure this effect, taking into account not only the survival of individuals introduced into the environment but also their conditions and reproductive ability [13,14,15,16,17,18,19]. There are also opinions that phenomena related to global warming will require a change in the approach to nature conservation, necessitating the use of completely new and unconventional methods [20].

Poland is located in a warm, temperate, transitional climate. The average annual temperature ranges from 6 °C to 8 °C, while the annual precipitation ranges from 630 mm to 700 mm [21]. The analysis of data from meteorological surveys carried out since the 1960s shows a significant increase in the number of hot days, the duration of heat waves, and the number of tropical nights. An increase in the annual average temperature has also been observed. Climate models predict an increase of 1 °C in the mean annual temperature in the years 2021–2050 and as much as 2 °C to 4 °C in the years 2071–2100. More frequent periods of drought are also predicted, as well as more days with intense precipitation. Progressive climate change and the accompanying sudden atmospheric phenomena can have serious consequences and lead to several disruptions in the natural ecosystems [22,23].

There are approximately 30 species of willow in the vascular flora of Poland, of which the downy willow (*Salix lapponum* L.) is particularly noteworthy. The natural range of this species includes the Arctic Circle in northern Eurasia and the Scandinavian Peninsula. In Poland, Scotland, as well as in some mountainous regions of central Europe, the species occurs outside its geographical range and is considered a glacial relict [24]. Natural populations of this species have been declining drastically since the 1950s, as confirmed by research carried out in the Polesie Lubelskie region, where up to 80% of the individuals have disappeared [25]. In the Krkonoše Mountains (on both the Czech and Polish sides), 21 *Salix lapponum* sites were recorded, and the populations studied were relatively stable [26]. In Poland, the Czech Republic, Ukraine, and Lithuania, the downy willow has the status of threatened with extinction [24]. One of the reasons for the decline of *Salix lapponum* populations is the changes occurring in natural ecosystems. Increased anthropopressure and changes in water relations have contributed to the degradation of peatlands, with which the species is closely associated. Downy willow is a small shrub, growing up to 1 m in height, and is distinguished by its elliptical gray–green leaves, which are covered with dense tomentum. It is mainly found in peatlands, preferring sunny and partly shaded sites with an acidic substrate (pH 4–6), rich in organic matter [27,28].

Due to the threat of complete extinction of the species in its isolated locations outside the center of the continuous range, since the early 2000s, several studies have been conducted on the biology [29,30,31] and ecology of the species [25,32,33,34,35,36], which helped collect the necessary information to prepare a procedure and carry out the first active protection measure for the species [37,38,39]. During their implementation, further questions arose, primarily concerning the prospects and conditions for the success of the species translocation in the context of global climate warming. Therefore, it has become important to check whether plants developed under ex situ conditions can adequately respond physiologically to environmental stresses, especially at early stages of growth. Interference in the resources of the populations of species threatened with extinction, e.g., collecting fragments of plant material, its propagation, and ex situ cultivation, requires the use of methods that eliminate the loss of valuable plant material both in the laboratory and after planting in natural sites [40,41]. This is extremely important due to the fact that these species exist in small and often dispersed populations.

In 2023, experiments were carried out to assess the condition of *Salix lapponum* individuals and their physiological response to short-term thermal stress during plant acclimatization in laboratory conditions. To verify this, controlled conditions were used to simulate phenomena that intensify in the natural environment due to climate change, i.e., sudden, short-term, and extreme decrease or increase in ambient temperature, which may affect the plants reintroduced to their natural sites.

## 2. Materials and Methods

### 2.1. Plant Material and Experimental Design

The plant material used in the present experiment was *Salix lapponum* plantlets obtained by micropropagation from vegetative parts of mother plants growing in natural populations in eastern Poland [18].

A total of 300 plantlets were transferred from in vitro conditions into soil substrate (peat–deacidified peat–sand–perlite 1:1:1:1 *v*/*v*) and placed in a phytotron, where they further grew under the same conditions of temperature (day/night 22 °C/20 °C), humidity (60%), and light (35 μmol·m^−2^·s^−1^ with the 16/8 h light/dark cycle).

After 1 week (Term 1), 5 weeks (Term 2), or 9 weeks (Term 3), 90 plants were selected randomly and placed for 12 h in a climatic chamber (MLR-352H-PE, PHCbi, PHC Europe, Breda, The Netherlands) under conditions of constant humidity (75%) and light intensity (53 μmol·m^−2^·s^−1^) but at different temperatures. Thirty plants were placed at a temperature of 0 °C (Variant 1) and thirty plants at 30 °C (Variant 2). The control group comprised 30 plants exposed to a temperature of 22 °C (Variant 3). Therefore, there were 9 different combinations of periods of growing plants in substrate (called terms) and temperature.

**Figure 1:**
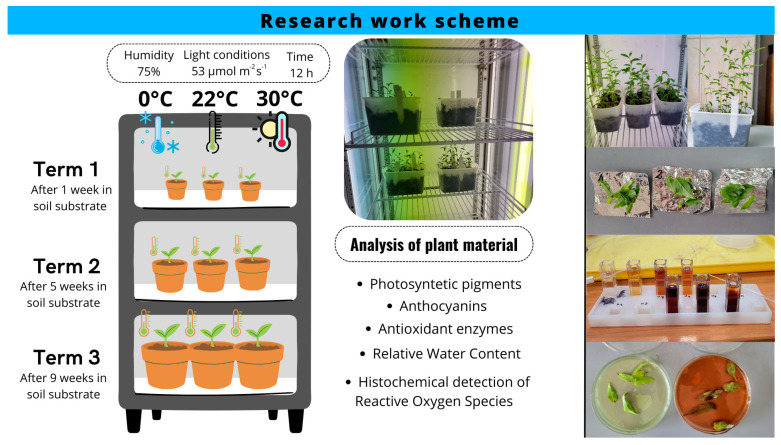
Study scheme, including timing, conditions of experiments, and analysis of plant material.

Immediately after 12 h, the plant material was subjected to laboratory analyses to determine the values of selected morphophysiological indicators and to assess the condition and response of the plants to thermal stress (Figure 1).

### 2.2. Determination of the Photosynthetic Pigment Content

The content of photosynthetic pigments, such as chlorophyll a, chlorophyll b, and carotenoids, was determined in fresh leaves collected from the middle part of the shoots from each combination. For the extraction of photosynthetic pigments, the leaves were homogenized in a 96% ethanol solution and then transferred to test tubes. The samples were placed in a water bath at 70 °C for 5 min and centrifuged (5 min, 10,000 rpm) to separate the liquid and solid phases. The absorbance of the obtained extracts was measured using a spectrophotometer (SPECORD 40, Analytik Jena, GmbH, Jena, Germany) at wavelengths of 470, 649, and 665 nm. The concentrations of the assimilatory pigments were calculated using a modified method proposed by Lichtenthaler and Wellburn [42].

### 2.3. Determination of the Anthocyanin Content

The anthocyanin content in the plant material was determined using the method described by Martinez and Favret [43]. An amount of 100 mg of leaves from each combination were cut into pieces and placed in test tubes containing methanol and HCl (in a 99:1 ratio). Then, the samples were stored in a refrigerator at 4 °C for 24 h. After the extraction process, the mixture was centrifuged (5 min, 10,000 rpm), and the obtained supernatant was used for spectrophotometric analyses. The absorbance of the extracts was measured at wavelengths of 527 and 652 nm.

### 2.4. Oxidative Stress Indicators

#### 2.4.1. Antioxidant Enzyme Activity

Leaf samples were collected from the middle part of the shoots. An amount of 100 mg of leaves from each combination was homogenized in 1 mL of 0.05 M phosphate buffer at pH 7.0 to determine the activity of guaiacol peroxidase (GPOX, EC 1.11.1.7) and catalase (CAT, EC 1.11.1.6). The prepared homogenates were centrifuged (10 min, 10,000 rpm, 4 °C). Enzyme activities were determined spectrophotometrically using a SPECORD 40 spectrophotometer (Analytik Jena GmbH, Jena, Germany). Catalase activity was measured at a wavelength of 240 nm, following the method described by Aebi [44]. For GPOX activity, the procedure of Małoplesza and Urbanek [45] was applied, with absorbance measured at 480 nm over 4 min at 1 min intervals.

#### 2.4.2. Histochemical Detection of Reactive Oxygen Species (ROS)

The accumulation of hydrogen peroxide (H_2_O_2_) and superoxide anion (O_2_^−^) in the leaf tissues of *Salix lapponum* was assessed using the qualitative method described by Kumar et al. [46]. Leaves were collected from the middle part of the shoots. H_2_O_2_ detection was performed using 3,3′-diaminobenzidine (DAB). The leaves were immersed in a DAB solution (1 mg/mL) for 10–12 h and then incubated in darkness at room temperature. After incubation, the leaves were briefly treated with boiling water, followed by hot 96% ethanol to remove chlorophyll from the tissues and reveal the brown polymerization product of DAB formed in the presence of H_2_O_2_.

To visualize the superoxide anion (O_2_^−^), nitroblue tetrazolium chloride (NBT) was used. The oxidized form of NBT (yellow) is reduced in the presence of endogenous O_2_^−^ to form blue–purple diformazan. Leaves were incubated in a 0.2% NBT solution in 50 mM phosphate buffer (pH 7.5) for 10–12 h in darkness. Subsequently, the tissues were decolorized in hot 80% ethanol until the blue–purple reduction product of NBT became distinctly visible.

The above-described analyses for ROS detection were performed only on *Salix lapponum* Term 3 plants (grown in soil substrate for 9 weeks). Those analyses could not be conducted on plants at earlier growth stages (Terms 1 and 2) due to the small surface area and delicate structure of their leaves.

### 2.5. Determination of Relative Water Content

The relative water content (RWC) in leaf tissues was determined according to the method described by González and González-Vilar [47]. Whole leaves were collected immediately after the treatment to measure the fresh weight (FW). They were placed in distilled water for 24 h to achieve full turgor. After that, the leaves were weighed, with excess surface water gently removed using blotting paper, to determine the turgor weight (TW).

In the final step, the leaves were placed in a drying oven (SML 32/250, Zalmed, Warsaw, Poland) at a temperature of 60 °C for 24 h and then weighed to determine the dry weight (DW). The relative water content was calculated using the following formula:

RWC = (FW − DW)/(TW – DW) × 100%

### 2.6. Statistical Analysis

Statistical analysis was performed for the photosynthetic pigments, anthocyanins, RWC, and antioxidant enzymes using two-way analysis of variance followed by Tukey’s multiple comparisons. All significant values are reported at *p* < 0.05. Principal Component Analysis (PCA) was employed to describe the structure of the data and identify the main factors differentiating the analyzed parameters. All computations were carried out in R environment, version 4.3.3 [48].

## 3. Results

### 3.1. Photosynthetic Pigment Content

**Figure 2:**
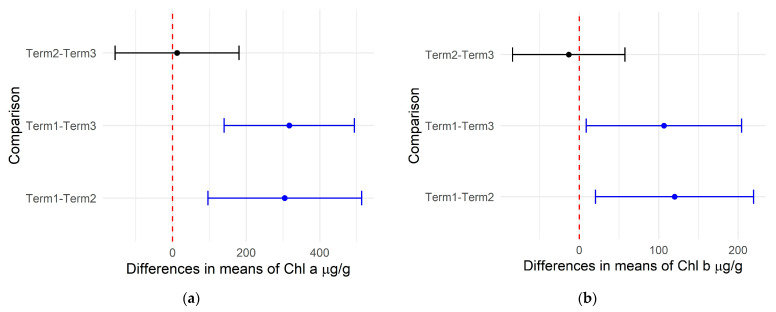

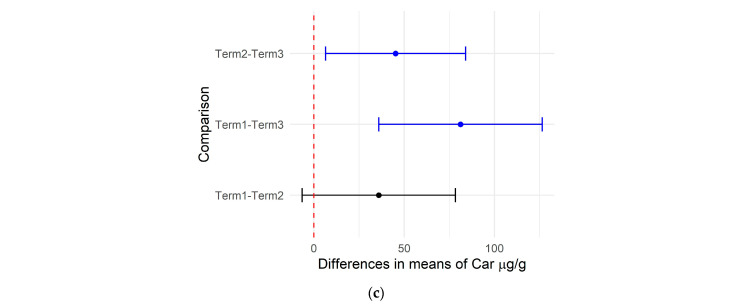
Differences in mean photosynthetic pigment contents: chlorophyll a (**a**), chlorophyll b (**b**), and carotenoids (**c**) in *Salix lapponum* tissues across three growth terms. Statistically significant differences (*p* < 0.05) are highlighted in blue, and non-significant differences are shown in black.

The content of photosynthetic pigments (chlorophyll a and b) in the leaves of the analyzed *Salix lapponum* plants did not differ statistically between the experimental variants on any of the three experimental terms. However, significant changes were observed in the content of total photosynthetic pigments in the plant materials between Term 1 and Term 2 and between Term 1 and Term 3 (Figure 2a,b). The analysis of the content of carotenoids in *Salix lapponum* tissues also revealed no significant statistical differences between their concentrations in plants exposed to temperature extremes for 12 h within each of the three experimental terms. The total carotenoid content of the plant material was statistically different between Terms 2 and 3 and between Term 1 and Term 3 (Figure 2c). The mean values of the photosynthetic pigments are presented in Supplementary Materials (Table S1).

### 3.2. Content of Anthocyanins

**Figure 3:**
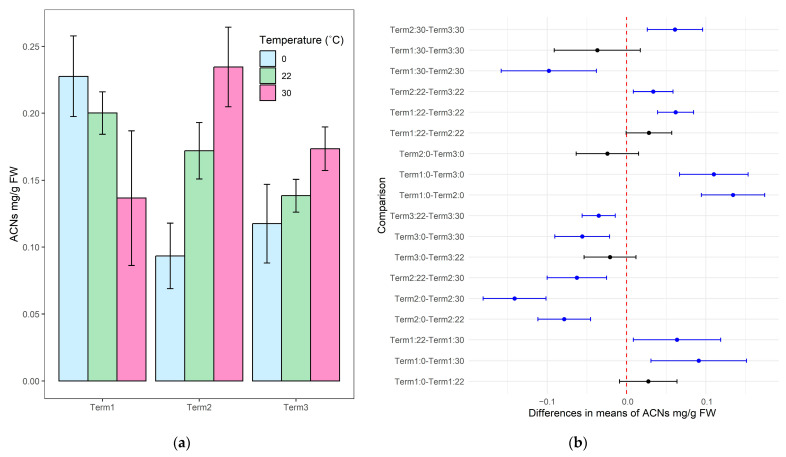
Anthocyanin content (ACNs) in the tissues of *Salix lapponum* exposed to different temperature extremes across three growth stages (Terms 1–3). Data are presented as mean ± standard deviation with a sample size of *n* = 4 (**a**). Pairwise comparison of ACNs between experimental conditions for *Salix lapponum*, showing statistically significant differences between term-temperature treatments across growth stages. Blue points indicate significant differences (*p* < 0.05), and black points indicate non-significant differences (**b**).

In the case of plants grown in soil substrate for 1 week (Term 1), higher anthocyanin levels were observed after 12 h of exposure to 0 °C compared to the control sample maintained at 22 °C. For plants exposed to 30 °C, significantly lower anthocyanin concentrations were noted in plant tissues, both compared to the control sample and to plants exposed to low temperatures. For plants cultivated in the phytotron for 5 weeks, statistically significant differences were observed between anthocyanin content in plants exposed to 0 °C and 30 °C. Concerning the control sample, the plants reached lower values for this indicator after 12 h at low temperature, while high temperature resulted in an increase in the anthocyanin content in the tissues of the tested specimens. A similar trend to that of plants from Term 2 was demonstrated by plants after 9 weeks of growth (Term 3), except that in this case, there were no significant differences between the anthocyanin content of the plants exposed to the temperature extremes (Figure 3). The mean values of the anthocyanin content in leaves are presented in Supplementary Materials (Table S1).

### 3.3. Antioxidant Enzyme Activity

**Figure 4:**
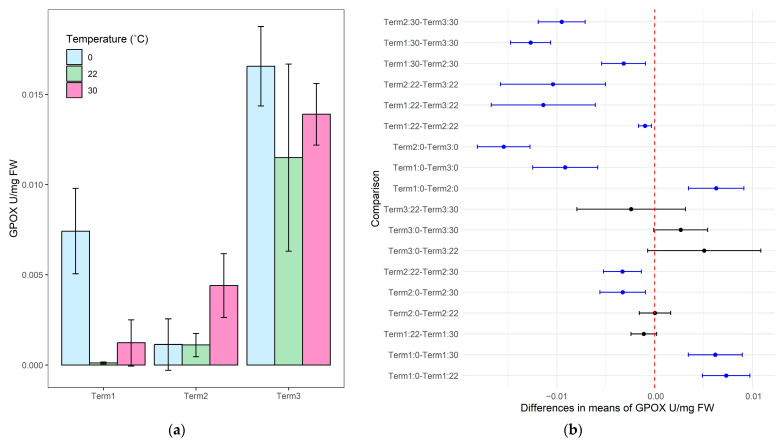
Guaiacol peroxidase activity (GPOX) in the tissues of *Salix lapponum* exposed to different temperature extremes across three growth stages (Terms 1–3). Data are presented as mean ± standard deviation with a sample size of *n* = 4 (**a**). Pairwise comparison of GPOX between experimental conditions for *Salix lapponum*, showing statistically significant differences between term-temperature treatments across growth stages. Blue points indicate significant differences (*p* < 0.05), and black points indicate non-significant differences (**b**).

The results of the guaiacol peroxidase activity analyses in plants grown for 1 week in the soil substrate indicated a significant increase in the content of that enzyme after 12 h of exposure to low temperature in comparison to the control (22 °C), in which the enzyme remained virtually inactive (Figure 4a). High temperature exposure resulted in a slight increase in guaiacol peroxidase activity. Experiments conducted on plants grown for 5 weeks revealed that high temperature induced a significant increase in peroxidase activity. The highest enzymatic activity of guaiacol peroxidase was observed in the last term of the study. Both low- and high-temperature treatments caused an increase in its activity. However, it was not significantly higher compared to the control sample at that experimental term. Comparing with the first and second test terms, the enzymatic activity of peroxidase in the third term was statistically higher in each experimental variant tested (Figure 4b). Mean values for the guaiacol peroxidase activity are presented in Supplementary Materials (Table S1).

Catalase activity was not detected in any of the combinations during the three study dates.

### 3.4. Histochemical Detection of Reactive Oxygen Species

**Figure 5:**
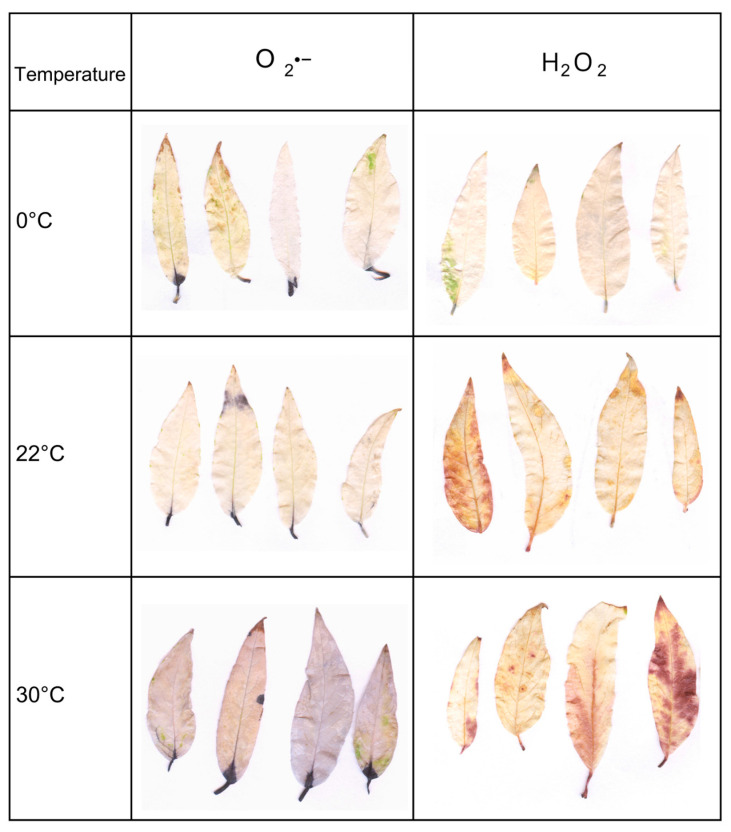
Visualization of reactive oxygen species (ROS) in *Salix lapponum* tissues exposed to different temperatures in the third term of the experiment.

*Salix lapponum* leaf tissues were subjected to histochemical detection of hydrogen peroxide and superoxide anion after nine weeks of growth in soil substrate. Plants in response to temperature extremes (0 °C and 30 °C) showed differential accumulation of reactive oxygen species (Figure 5). Plants exposed to low temperatures (0 °C) revealed only trace amounts of H_2_O_2_ staining product visible on the leaves. In the control sample (22 °C), the staining intensity of the plant material was moderate, with a brown staining product appearing on the entire leaf surface. When the plants were exposed to high temperatures (30 °C), the leaf staining was the strongest, and it was visible on most of the surface of the tested leaves. Visualization using the NBT reagent indicated an increased superoxide anion content after exposure of plants to 30 °C, which was evident by the intense coloring of the entire leaf blade surface. In the low-temperature treatment and control, the dark color, which is a product of the reaction, indicates the accumulation of superoxide anion only within the petioles.

### 3.5. Relative Water Content (RWC)

**Figure 6:**
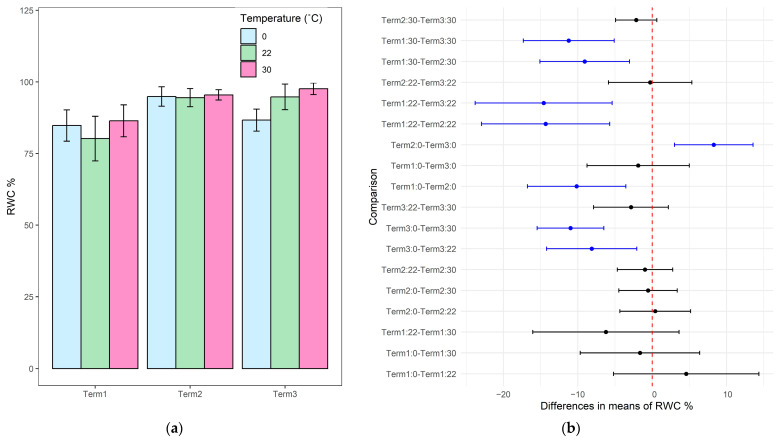
Relative water content (RWC) in the tissues of *Salix lapponum* exposed to different temperature extremes across three growth stages (Terms 1–3). Data are presented as mean ± standard deviation, with a sample size of *n* = 4 (**a**). Pairwise comparison of RWC between experimental conditions for *Salix lapponum*, showing statistically significant differences between term-temperature treatments across growth stages. Blue points indicate significant differences (*p* < 0.05), and black points indicate non-significant differences (**b**).

The relative water content of the tissues of *Salix lapponum* plants that had been exposed to temperature extremes for 12 h was investigated. Regarding Term 1, there was an increase in the RWC after both 0 °C and 30 °C exposure compared to the control (22 °C), although those differences were not statistically significant. While conducting tests on plants growing for 5 weeks in soil, the relative water content was high in all combinations, but the changes induced by both low and high temperatures compared to the control sample were minimal. Only after 9 weeks of growth in soil substrate, there was a significant decrease in RWC in tissues of plants exposed to low temperature, compared to the control sample. A significant increase in the relative water content was also found between Term 1 and Term 3 after exposure to 30 °C, between Term 1 and Term 2 after exposure to 0 °C, and between Term 1 and Terms 2 and 3 after exposure to 22 °C. In Term 2, a significantly higher RWC value was recorded at low temperature compared to Terms 1 and 3 (Figure 6). Mean values of the RWC content are presented in Supplementary Materials (Table S1).

### 3.6. Principal Component Analysis (PCA)

Principal component analysis (PCA) was used to determine the relationship between the studied indicators of the physiological response of *Salix lapponum* to temperature extremes. All variants of the experiment carried out were included; these included the following parameters: Chl a, Chl b, Car, ACNs, GPOX, and RWC. The first three components explain 91.2% of the total variation in the data. The first component, PC1, explains 52.3% of the total variance; the second, PC2, 20.3%; and the third, PC3, 18.6%. Since the two components together explain 72.6% of the total variance, it can be concluded that the PCA plot reflects the variability of the data well and that the two components are suitable for understanding the main patterns of plant responses to heat stress simulated in the experiments. The first axis, PC1, is strongly associated with Chl a, Chl b, and carotenoids, suggesting that these variables have the greatest influence on the differences in the data. The second axis, PC2, is related to GPOX and RWC. The third axis, PC3, is associated with ACNs, suggesting that this variable also has a significant, although smaller, effect on the differences in the data.

We observe a very strong positive correlation for Chl a, Chl b, and Car, a weaker but still positive correlation between this group of variables and GPOX, and a negative correlation between GPOX and ACNs. It can also be noted that the correlation between RWC and both GPOX and ACNs is close to zero.

**Figure 7:**
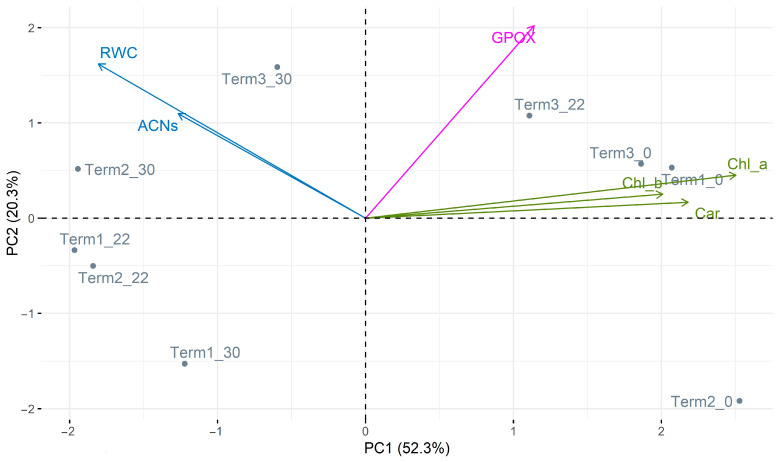
Results of the principal component analysis (PCA) of selected parameters of *Salix lapponum*’s physiological response to temperature extremes (ACNs—anthocyanins, GPOX—guaiacol peroxidase, Chl a—chlorophyll a, Chl b—chlorophyll b, Car—carotenoids, RWC—Relative Water Content).

The spatial distribution of the analyzed experimental cases (Term) in the PCA plot provides further insight into the physiological responses of *Salix lapponum* to temperature extremes. Plants exposed to low temperatures (0 °C, such as Term1_0 and Term3_0) showed higher than average levels of Chl a, Chl b, Car, and GPOX. These results suggest that these parameters play a key role in plant adaptation to cold stress. In contrast, plants exposed to higher temperatures (30 °C, e.g., Term1_30 and Term3_30) showed higher average RWC and ACN values. This indicates a change in the physiological priorities of the plant under heat stress, with increased water content (RWC) and anthocyanin production (ACNs) potentially contributing to the tolerance to higher temperatures (Figure 7).

## 4. Discussion

Thermal stress associated with global warming and heat waves is one of the main factors limiting crop production, leading to reduced plant biomass. In response to unfavorable environmental conditions, plants have developed several defense mechanisms to protect themselves from the effects of stress [6,9]. There is a wide range of biochemical and physiological indicators of plant condition that can be monitored in the context of response to environmental stresses, which allows for the assessment of plant adaptive strategies at different stages of growth and development [49].

Photosynthetic pigments are sensitive to high temperature stress, which can lead to their degradation and reduced photosynthetic efficiency [50]. Under the influence of thermal stress caused by high temperatures, chloroplasts undergo serious changes leading to structural damage [51]. In the current study conducted on *Salix lapponum*, no significant changes in chlorophyll content were observed after 12 h exposure to temperatures of 30 °C and 0 °C. A short exposure to elevated temperatures may not have been sufficient to induce visible changes in the levels of photosynthetic pigments in the plant tissues, as in the study conducted by Wassie et al. [52] on alfalfa (*Medicago sativa* L.), it was shown that after a 7-day exposure to high temperatures, the content of photosynthetic pigments decreased significantly, suggesting a strong stress response in that species. Furthermore, in the study conducted on *Salix lapponum,* the only significant changes observed were the differences in pigment content between subsequent study dates, which most likely reflect natural changes related to the plant’s development and not a response to short-term thermal stress.

Anthocyanins play an important antioxidant function and have a crucial role in scavenging reactive oxygen species, protecting plants from a wide range of damages caused by environmental stress [53]. Studies conducted on *Fagopyrum tataricum* showed an increase in anthocyanin synthesis in response to low-temperature stress. A positive correlation was also demonstrated between the antioxidant capacity and anthocyanin accumulation in *Fagopyrum tataricum* tissues, which potentially protected sprouts from low temperatures [54]. The significant role of anthocyanins in neutralizing ROS and acclimatization to low temperatures may be evidenced by the fact that plants deprived of these compounds show a higher level of reactive oxygen species accumulation in tissues under oxidative stress induced by low temperature [55,56]. The protective role of anthocyanins was also demonstrated in the case of high-temperature stress. Studies conducted in *Arabidopsis* showed severe damage to cell membranes in anthocyanin-deficient individuals, while in wild-type individuals, the damage was much lower [57]. With regard to the experiments presented, *Salix lapponum* plantlets demonstrated similar responses to the stress induced by temperature extremes. Plants exposed to extreme temperatures at the initial stage of growth in the phytotron (Term 1) contained significantly more anthocyanins when exposed to low temperatures compared to the control (22 °C) or increased temperatures (30 °C), which indicated the intensification response to stress in low temperature conditions [58]. The opposite trend in the second and third terms of the experiment may indicate the activation of defense mechanisms in response to heat stress [57]. In the last term of the research, after 9 weeks of plant growth in the soil substrate, the fluctuations in the anthocyanin levels concerning the control samples were lower than at the earlier stages of growth, which might indicate that in this particular phase of growth, the plant has achieved a certain level of adaptation to heat and cold stress. This adaptation may also be the result of other protective mechanisms that were activated over time. This may be indicated by a significant increase in guaiacol peroxidase activity after the exposure of downy willow plants to both low and high temperatures in the last term of the study (Term 3).

In addition to non-enzymatic antioxidants, which include, among others, anthocyanins, specialized antioxidant enzymes play an important role in protecting the plants from the adverse effects of stress. These enzymes contribute to maintaining plant homeostasis by reducing excess ROS [59]. In the case of plants from the in vitro cultures, lower levels of antioxidant enzymes, such as catalase and peroxidase, are observed due to constant, controlled growth conditions. Unlike the natural environment, where plants are exposed to variable abiotic stresses, phytotron conditions are stable and devoid of stress factors, such as temperature fluctuations or variable humidity, which can naturally stimulate the activity of defense mechanisms. Consequently, the lack of environmental stressors leads to lower activation of antioxidant enzymes in the in vitro cultures [60,61]. This may be reflected in the results of guaiacol peroxidase activity in downy willow tissues exposed to extreme temperatures obtained in Terms 1 and 2 of the experiment. Despite statistically significant differences in the GPOX activity (compared to the control sample), in the case of low temperature in Term 1 and high temperature in Term 2, its content in *S. lapponum* tissues was relatively low. In the conducted experiment, no catalase activity was detected in response to thermal stress. However, this enzyme is widely used as an indicator of antioxidant responses in studies on the response of plants to abiotic stress because it plays a key role in neutralizing hydrogen peroxide, one of the main reactive oxygen species formed during stress. Numerous reports suggest that the presence and activity of catalase can be regulated by other peroxidases, such as ascorbate peroxidase and guaiacol peroxidase, which may limit or change its activity depending on the type of stress and the specific need of the plant for hydrogen peroxide detoxification [6,62,63].

Considering that the histochemical method of detecting hydrogen peroxide and superoxide anion is a qualitative technique that does not provide detailed quantitative data on the concentration of these reactive oxygen species [64] and the fact that in the study on *Salix lapponum* individuals presented in this article, it was possible to visualize ROS only in the third experimental term, the presented images should be regarded as a supplementary, indicative tool. The analyses of ROS accumulation in *Salix lapponum* tissues were also carried out in a separate study by Pogorzelec et al. [38], which aimed at assessing the condition of the plant in different stages of acclimatization. Significant differences in the intensity of leaf color were found depending on the stage of acclimatization, from laboratory conditions to the harsh conditions of natural ecosystems. In laboratory conditions where environmental stress was absent, no clear ROS staining products were observed. However, when the plants were transferred to a field acclimatization station, where they were exposed to rapid changes in environmental conditions, intense staining indicated a high level of oxidative stress. In the studies described in this article, the most pronounced staining was observed as a result of exposing the plants to high temperatures. However, short-term stress under controlled phytotron conditions did not cause such a pronounced accumulation of ROS as seen in the long-term exposure to various stress factors during acclimatization in a field station [38].

Relative water content (RWC) is a commonly used indicator reflecting hydration and general plant condition, especially important in abiotic stress, such as extreme temperatures or drought [65]. The results of the experiment conducted on *Salix lapponum* plants reveal a tendency of the plants to increase the RWC in response to high temperatures in all experimental terms, especially in Term 3, where RWC reached almost 98%, indicating the ability of the tested plants to retain water under high temperature conditions. The decrease in RWC in *S. lapponum* tissues in the last term after exposure to 0 °C compared to the control suggests a reduced capacity for water retention in older plants under cold stress [66,67]. However, the consistently high RWC values throughout the experiment indicate the good physiological condition of the plants, and the observed differences in the RWC values may come from individual traits.

To sum up, the results of the conducted research demonstrated that *Salix lapponum* individuals exhibit different physiological reactions to thermal stress depending on the duration of their growth in soil substrate. In general, plants at early growth stages responded to stress induced by low temperatures, while older plants were more sensitive to high temperatures, as evidenced by higher anthocyanin levels and increased activity of guaiacol peroxidase. Short-term exposure to extreme temperatures did change the content of chlorophyll a and b in plant tissues. It was found that catalase activity is a non-indicative marker of stress in the case of the species studied in the conducted experiments.

## 5. Conclusions

The obtained results presented suggest that *S. lapponum* plants from ex situ cultivation can survive periods of short-term thermal stress because they are capable of activating a number of physiological defense mechanisms. The studied indicators of plant response to stress (e.g., anthocyanins, guaiacol peroxidase, or RWC) can be used to monitor the plant condition throughout the entire process of their translocation. However, it is still unclear how the frequent occurrence of such stress factors will affect the plants’ condition and acclimation capacity. Undoubtedly, there is a need for continued research that will take into account longer periods of exposure to extreme temperatures, as well as the impact of low and high temperature fluctuations on the plants of *S. lapponum*.

The conservation of endangered plant species, such as *S. lapponum,* in the face of global warming and climate change will soon need to rely not only on standard active conservation measures but also on a thorough understanding of the plant physiological response patterns under stress conditions. Such knowledge will enable the modeling and optimizing of methods using species translocations, taking into account possible scenarios of changes occurring in the substitute habitats.

## Data Availability

Data supporting the findings of this study are available from the corresponding author upon reasonable request.

## Supplementary Materials

The following supporting information can be downloaded at https://www.mdpi.com/article/10.3390/biology14010019/s1: Table S1: Mean values of the tested parameters of the physiological response of *Salix lapponum*.
