# Supplementary material for: Do Endangered Glacial Relicts Have a Chance for Effective Conservation in the Age of Global Warming? A Case Study: Salix lapponum in Eastern Poland
Source: Biology (Basel). 2024 Dec 28;14(1):19. doi: 10.3390/biology14010019 (PMC11763092; doi:10.3390/biology14010019)
Supplement: Supplementary file 1 [file biology-14-00019-s001.zip › biology-3363996-supplementary.pdf]

Table S1. Mean values of the tested parameters of the physiological response of *Salix lapponum.*

Parameter	Term 1			Term 2			Term 3		
	0°C	22°C	30°C	0°C	22°C	30°C	0°C	22°C	30°C
Chlorophyll a (µg/g FW)	1928.73	1884.08	1788.70	1439.03	1451.26	1802.46	1476.52	1540.31	1646.77
Chlorophyll b (µg/g FW)	829.18	684.03	617.62	548.41	545.46	669.15	612.38	579.30	612.36
Carotenoids (µg/g FW)	293.49	334.19	325.27	272.44	261.70	316.01	216.91	238.31	261.11
Anthocyanins (mg/g FW)	0.2276	0.2002	0.1366	0.0934	0.1720	0.2346	0.1176	0.1384	0.1735
Guaiacol peroxidase (U/mg FW)	0.007	>0.001	0.001	0.001	0.001	0.005	0.0018	0.0014	0.0015
Relative Water Content (%)	84.77	80.19	86.41	94.92	94.51	95.47	86.65	94.78	97.62
